# Use of Post-mortem Brain Tissue in Investigations of Obsessive-Compulsive Disorder: A Systematic Review

**DOI:** 10.2174/1570159X21666230829145425

**Published:** 2023-08-31

**Authors:** Christine Lochner, Petrus J.W. Naudé, Dan J. Stein

**Affiliations:** 1 SA MRC Unit on Risk and Resilience in Mental Disorders, Department of Psychiatry, University of Stellenbosch, Stellenbosch, South Africa;; 2 Department of Psychiatry and Mental Health & Neuroscience Institute, University of Cape Town, Cape Town, South Africa;; 3 SA MRC Unit on Risk and Resilience in Mental Disorders, Department of Psychiatry and Mental Health & Neuroscience Institute, University of Cape Town, Cape Town, South Africa

**Keywords:** Obsessive-compulsive, post-mortem, systematic review, neurocircuitry, neurochemistry, neuroimaging, fronto-striatal circuitry

## Abstract

**Background:**

Post-mortem examination of the brain is a key strategy to increase our understanding of the neurobiology of mental disorders. While extensive post-mortem research has been undertaken on some mental disorders, others appear to have been relatively neglected.

**Objective:**

The objective of the study was to conduct a systematic review of post-mortem research on obsessive-compulsive disorder (OCD).

**Methods:**

A systematic review was performed in accordance with PRISMA guidelines to provide an overview of quantitative, qualitative, or mixed methods primary research studies on OCD. Search platforms included NCBI Pubmed, SCOPUS, and Web of Science.

**Results:**

A total of 52 publications were found, and after the removal of works not meeting the inclusion criteria, six (6) peer-reviewed publications remained. These post-mortem studies have provided data on DNA methylation, cellular and molecular alterations, and gene expression profiling in brain areas associated with OCD.

**Discussion and Conclusion:**

Included studies highlight the potential value of post-mortem brains from well-characterized individuals with OCD and suggest the need for additional work in this area.

## 1 INTRODUCTION

Obsessive-compulsive disorder (OCD) is a chronic, severely debilitating neuropsychiatric condition characterized by repetitive, intrusive thoughts (obsessions) and/or repetitive mental acts or behaviors (compulsions). In DSM-5 and ICD-11, OCD is placed under a category of obsessive-compulsive and related disorders, in line with evidence of significant overlaps in the phenomenology and psychobiology of these conditions [1-3]. OCD is also one of the most prevalent and disabling neuropsychiatric disorders. The National Comorbidity Survey Replication found a lifetime prevalence of 2.3% [4], with other epidemiological studies indicating a lifetime prevalence of 1.9% to 3.0% [5]. In a recent meta-analytic review, the overall aggregate current and lifetime OCD prevalence estimates were 1.1% and 1.3%, respectively [6]. In the Global Burden of Disease (GBD) study, anxiety disorders, including OCD, have been ranked as the sixth largest global contributor to non-fatal health loss [7, 8]. In terms of functional impairment due to OCD, the GBD study emphasized significant income loss and decreased quality of life [9], and later work also suggested a substantial loss of productivity, with an average of 46 days absent from work in the last year due to OCD [4]. Established first-line treatment, consisting of a selective serotonin reuptake inhibitor (SSRI) and/or cognitive behavioral therapy, including exposure and response prevention (CBT/ERP), is efficacious for approximately half of the participants with OCD [10, 11], with non-response to treatment being associated with lack of adherence to treatment, poor insight, comorbid depression, and increased OCD severity, but also serious social disability, an elevated suicide rate, and major healthcare expenditures [12, 13]. Improving treatment by incorporating information on neurobiology [14] may be one strategy to lower these burdens. Most of what we know about the pathophysiology of OCD has emerged from animal models of OCD and from neuroimaging and pharmacological studies on humans with this condition. Animal models of stereotyped behaviours and grooming reminiscent of some OCD in rodents and primates [15, 16] have highlighted the importance of fronto-striatal circuitry, as well as the serotonin, dopamine, and glutamate neurotransmitter systems [17-25] (For comprehensive reviews, see Ahmari and Chamberlain *et al*. [26, 27]). In humans, multiple studies have supported a significant genetic contribution to OCD, as shown by twin and family aggregation studies suggesting higher concordance rates in monozygotic *versus* dizygotic twins [28], and population-based studies confirming substantial heritability in OCD with the risk for OCD among relatives increasing according to the degree of genetic relatedness to the proband [29, 30]. Consistent with relevant animal work, results from candidate gene and genome-wide association studies (GWAS), as well as pharmacological challenge and pharmacotherapy studies, suggest that the serotonergic, dopaminergic, and glutamatergic systems are involved in OCD [31-35]. GWAS have also shed light on the genetic architecture of OCD, suggesting the heritability of OCD on the basis of common SNPs (*e.g*., SNPs within *DLGAP1*) [36]. Rare variants, including cytogenetic abnormalities and copy number variants (CNVs) in genes or loci, such as *PTPRD*, *BTBD9*, *NRXN1*, *ANKS1B*, and 16p13.11, have also been implicated in the pathogenesis of OCD [37]. Still, identification of the specific genetic determinants requires further investigation in larger samples. Moreover, OCD is a multidimensional disorder with multiple symptom dimensions that may each have distinct aetiological origins [31, 38] and distinct neural circuitry [39]. There may also be a range of additional neurotransmitters and neuropeptides implicated in OCD (human: [40, 41]; animal: [42, 43]). Advances in human brain imaging methods have also been important in furthering the field, giving impetus to models of OCD neurocircuitry [44]. Neuroimaging studies have suggested significant neurobiological changes in OCD, including alterations in volume, connectivity, and activity, involving several brain regions that comprise cortico-striato-thalamo-cortical (CSTC) circuits (involved in sensorimotor, cognitive, affective, and motivational processes) [45-47]. The prevailing pathophysiological model of OCD has emphasized increased activity in brain regions that form a CSTC loop [48-54]. This circuitry involves pathways from cortical brain regions, which include the orbitofrontal cortex (OFC) and the anterior cingulate cortex (ACC), to the thalamus *via* the striatum, possibly involving reduced inhibition of the thalamus and increased excitatory feedback to frontal brain regions [31]. To reduce or neutralize the distress resulting from obsessions, individuals with OCD perform repetitive and ritualistic behaviors that may involve striatal regions [53]. More recently, neuroimaging research undertaken by the ENIGMA consortium has also highlighted the involvement of cortical and subcortical alterations in OCD [55]. In addition to the role of CSTC circuitry, there is also growing evidence for the involvement of several other regions, such as the occipital, parietal, and cerebellar cortices [56-58], and other large-scale networks, including the Default Mode Network, the Fronto-Parietal Network, and the Salience Network [59-61]. Both efficacious pharmacotherapy and psychotherapy interventions may normalize CSTC alterations in OCD [47, 62-64], but other areas may also be impacted by treatment [57]. Studies on the involvement of inflammatory and immune pathways in OCD indicate an association with low-grade inflammation, neural antibodies, neuro-inflammatory, and auto-immune disorders [65]. It has also been suggested that neuroinflammatory/autoimmune theories of OCD should extend beyond the basal ganglia to include the CSTC circuit [66]. These investigations, which may include subset profiling in OCD, are preliminary [41, 67, 68], thus requiring further research. Despite advancing our extensive knowledge of OCD, these approaches have some limitations. For example, the validity of animal models has been questioned as they do not reflect the complexity of the human brain and because some symptoms associated with OCD, such as intrusive thoughts or images, or suicidality in some cases, cannot be simulated in animal models. In addition, although studies on humans that use peripheral blood can uncover genes and soluble proteins that are associated with the disorder being studied, the genetic blueprint determined in this way does not necessarily reflect the gene expression, protein levels, or functional changes in the brain [69], likely because of the intact blood-brain barrier typical of psychiatric disorders and the unique properties of brain tissue [70]. Furthermore, existing MRI strategies are not able to identify cellular and molecular alterations in the human brain, which could assist the search for and design of more effective treatment strategies. Post-mortem examination of the brain is a key strategy to overcome some of the limitations associated with other approaches and may contribute to our understanding of the underlying neurobiology of disorders, such as OCD. While there is a growing body of post-mortem work on mental disorders, such as schizophrenia, there seems to have been less attention to conditions like OCD. We aimed to conduct a systematic review of existing findings from post-mortem research on obsessive-compulsive symptomatology/OCD.

## 2 MATERIALS AND METHODS

A systematic review of the literature on post-mortem investigations in patients with obsessive-compulsive symptomatology/OCD was executed using the Preferred Reporting Items for Systematic Reviews and Meta-Analyses (PRISMA) statement [71]. The study protocol was registered with Open Science Framework (https://osf.io/zq4ju/).

### 2.1 Eligibility Criteria

Studies examining post-mortem brains of individuals of all ages and any sex and who had an established diagnosis of OCD or who presented with obsessive-compulsive symptoms during their lifetime were considered. This review considered studies conducted in any setting. Publications were only included if they were in English. In terms of comparators, articles were eligible only if the studies included suitably matched healthy individuals. In terms of the designs of the studies evaluated for inclusion, this review considered quantitative, qualitative, or mixed methods primary research studies reporting on post-mortem findings in OCD or obsessive-compulsive symptoms.

### 2.2 Information Sources and Search Strategy

Searches using NCBI Pubmed, SCOPUS, and Web of Science were conducted. The reference lists of relevant articles were scanned to retrieve additional studies to be included in the review. Search terms that were used for all platforms were: “obsessive-compulsive disorder” OR “obsessive-compulsive disorder” OR “obsessive-compulsive symptoms” OR “OCD” AND (“post-mortem” OR “post mortem” OR “postmortem”), OR (“obsessive-compulsive disorder” OR “obsessive-compulsive disorder” OR “obsessive-compulsive symptoms” OR “OCD”) AND (“post-mortem” OR “post mortem” OR “postmortem”) AND (“neuropathology” OR “neurocircuitry” OR “neurochemistry” OR “neuropeptide” OR “neurotransmitter” OR “neuroinflammation” OR “inflammation”). In this way, only the articles with data from original research pertaining to post-mortem investigations in OCD/obsessive-compulsive symptomatology were collected. Information on the use and significance of brain donation in psychiatry was obtained from the selected articles. There were no search restrictions on publication dates, and all articles published before or on 14^th^ September, 2022, which met inclusion criteria, were considered for inclusion.

### 2.3 Data Collection and Selection

Titles and abstracts of retrieved studies were logged in a reference management database after duplicates were deleted. The screening of titles and abstracts for eligibility was conducted by two co-authors independently (CL and DJS). There were no conflicts about the inclusion of any such records identified for full-text assessment in this screening process. A flowchart of the selection process and output is presented in Fig. (**1**) [71]. After removing non-relevant items based on study titles and exclusion of duplicates, the initial search rendered 210 records. After the exclusion of duplicates, 128 records remained. The abstracts of these potentially eligible records were subsequently reviewed. After the exclusion of records based on the eligibility criteria (*e.g*., exclusion of non-OCD, non-post-mortem, and animal work), a search was done to retrieve the full-text manuscripts of 8 items. We were able to retrieve the full-text manuscripts of six (6) original research articles. Two of the 8 records were available as an abstract only (a conference proceeding and another paper currently in press, respectively) and were thus excluded from the review (Table **1**).

### 2.4 Data Extraction

The following data were extracted from the selected full-text clinical research articles: Country of investigation, study design, sample size and population, diagnostic method, the brain regions that were investigated, and the main findings. The Appraisal tool for Cross-Sectional Studies (AXIS) was used to assess the study design and reporting quality of the included articles [72]. The 20-question AXIS appraises the quality of study design, sample size and characteristics, measures, internal consistency, results, analysis, ethical standards, and limitations of the investigations. There are three primary categories: quality of reporting (7 questions), study design quality (7 questions), and possible introduction of biases (6 questions). In this way, the AXIS provides a tool for raters to critique each part of a study to judge overall quality. For the present review, one rater (CL, a researcher/ clinician in the health sciences field) scored each study that was included in the review. For each, the 20 AXIS questions were scored by assigning numeric values to two categorical responses: “Yes” (scored as 1) and “No” (scored as 0). Items not applicable to the article being rated were scored as a “yes” (1). After each item was scored, the values of the 20 scores were summed for a total appraisal score value, indicating the level of methodological quality of the study. For the critical appraisal of articles included in this review, the rater predetermined that a publication with acceptable quality would obtain a total appraisal score equal to or exceeding 70% of the total (*i.e*., at least 14 out of 20 questions scored as 1).

## 3 RESULTS

The literature search yielded six scientific publications on post-mortem investigations of the brains of individuals that presented with OCD or OC symptomatology during their lifetime. The specifics of each article, including the citation, the country where the study was conducted, the source of the brain specimens, the study design, sample size and population, the methods used to diagnose the participants, and the main findings, are detailed in Table **1**.

## 4 DISCUSSION

The post-mortem studies included in this systematic review have provided data on DNA methylation, cellular and molecular alterations, and gene expression profiling in brain areas associated with OCD. The earliest of these by Jaffe and colleagues used post-mortem human brain tissue from individuals with “obsessive psychiatric disorders”, including OCD, eating disorders (EDs), such as anorexia nervosa and bulimia nervosa, tics, and obsessive-compulsive personality disorder (OCPD), attempting to assign a molecular function to clinical genetic risk [73]. They aimed to evaluate risk-associated genes and SNPs that have been previously reported for OCD, anorexia nervosa, and bulimia nervosa. In addition, they also aimed to identify genes that were differentially expressed by comparing OCD and ED patients with controls in the first post-mortem human brain sample of its kind. A third aim was to examine whether the risk variants could explain the differential expression through *cis* or *trans* genetic mechanisms. To achieve this, the researchers collected dorsolateral prefrontal cortex (DLPFC) tissue, *i.e*., Brodmann’s area (BA) 46 and part of granular frontal area 9, from cases (N = 133, comprising ED: *N* = 15, and OCD/OCPD or tics (OCD/OCPD/tics): *N* = 16), as well as from matched controls (N = 102). Total RNA was extracted from all samples [74], while DNA for genotyping was obtained from cerebellar tissue. Their findings suggested a significant association between a small subset of previously identified genetic risk variants for OCD and EDs and gene expression within healthy controls. Two of the six significant genes (PVALB and RFNG) differentially expressed in EDs were also significant in patients with OCD. Both PVALB-expressing interneurons and RFNG appear to be vulnerable to stressors and have recently been implicated in many neuropsychiatric diseases [75, 76] but not yet OCD. Gene expression in the DLPFC identified 286 genes differentially expressed in patients with OCD/OCPD/tics compared to controls. Although the SNPs that were examined could not explain the gene expression differences between the broad “obsessive” patient group (OCD/OCPD/tics and ED) and controls, the large number of differentially expressed genes in “obsessive” patients *versus* controls provided a set of candidate genes for further association studies in larger cohorts. The focus of the first comparative study that used post-mortem tissue from a sample of OCD cases and controls was relatively narrow, primarily addressing the inconsistent *in vivo* findings of the involvement of abnormalities in the OFC [77]. Although abnormalities in the OFC have been one of the most consistent findings in OCD [78], there are some structural imaging studies comparing OCD patients and controls that reported greater OFC grey matter volume [79-81], whereas others showed reduced volume [50, 82-86]) in patients. Functional MRI studies have also shown apparently conflicting results, with hyper- [87-89] and hypo-activation [48] both being reported. To address these remaining inconsistencies, De Oliveira and colleagues [77] studied OFC abnormalities at the cellular level in seven individuals with OCD after an extensive post-mortem clinical evaluation with their next of kin and seven matched control cases. This is the first cytoarchitectonic delineation of the OFC of post-mortem brains of OCD patients. Lamiar neuronal density and volume of the anteromedial (AM), medial orbitofrontal (MO), and anterolateral (AL) areas of the OFC were estimated. In terms of density, the results suggested the presence of statistically significant layer- and region-specific lower neuron densities in the OCD cases in the respective OFC areas, with the average neuron density being 21-25% lower in the OCD cohort. Their findings further showed that volumes of the OFC areas were similar between the OCD patients and controls. Notably, these findings concerning OFC volume were subject to histological artifacts, and therefore, equivocal concerning pathological volume may either increase or decrease in OCD. Similarly, findings from *in vivo* structural neuroimaging research have reported OFC volumetric alterations, including both increments and decrements in OCD patients [83, 90]. It was argued that these findings suggest complex layer and region-specific neuronal loss with preservation of the OFC volume by the proliferation of neurites and neutrophil increase in older patients with OCD (the average age of participants was 70+ years), which could have a considerable impact on information processing within OFC regions. Since the majority of cortical pyramidal cells are glutamatergic, De Oliveira and colleagues recommended further exploration of the correlation between laminar and region-specific neuron deficits with elevated glutamate and glutamine (Glx/Cr) in the OFC white matter of OCD patients, candidate gene association studies as well as the differential expression of the glutamate transporter genes *SLC1A1, SLITRK, DLGAP1,* and *GRIN2B* [77]. Also in 2019, Lisboa and colleagues from the same group of researchers published another post-mortem OCD study using unbiased RNA-sequencing (RNAseq) to compare the transcriptomes of 3 separate striatal subregions (putamen (PT), caudate nucleus (CN), and nucleus accumbens (NAC)) [91]. Their study was motivated by the hypothesis that three smaller circuitries from different striatal subregions encompassing the CSTC may play an important role in the pathophysiology of OCD [92, 93]. These three circuits have been validated using various modalities, including neuropsychological tests, gene expression work, and diffusion-weighted imaging (DWI) [94-96]. Their findings, in a cohort of six OCD patients and eight matched controls, suggested that differentially expressed genes and network connectivity deregulation were specific for each striatum region [91]. Moreover, some genes that showed an association with rare or common variation in large-scale OCD genomic studies were differentially expressed in specific region comparisons. As such, the data rendered by this study indicated striatum subregion specificity, confirming the so-called *tripartite* model of the striatum. The investigators argued that at the transcriptional level, their study supports differences in the 3 circuit CSTC model associated with OCD. Genes that were differentially expressed between OCD cases and controls were also found to be enriched for synaptic and immune function protein-encoding transcripts. It was thus suggested that there might be different biological processes specific to each striatum region, each contributing in various ways to OCD pathophysiology. Another study from the same research group subsequently focused on DNA methylation (DNAm) changes and gene expression in post-mortem brain tissues of the cortical (anterior cingulate gyrus and OFC) and ventral striatum (PT, CN, and NAC) areas from a slightly larger cohort of eight OCD patients and eight matched healthy controls [97]. Their investigation was in response to studies on DNAm in substitute peripheral tissues, such as blood [98-100], including mononuclear cells [101] and saliva [102], and involved consideration of the methylation clock in brain tissues from patients with OCD as a complement of tissue senescence, associated with physiological ageing [103]. De Oliveira and colleagues found no differentially methylated CpG (cytosine-phosphate-guanine) sites (DMSs) in any of the brain areas that were investigated, but there was a predominantly hypomethylation pattern in all brain regions (except the NAC), especially those involved with genes related to synaptic plasticity and the immune system [97]. Moreover, they found older DNAm age and higher aging trends regarding age acceleration (AA) difference and AA residuals for all areas except the ACC (for the OCD group). Their findings suggest a role for cellular communication, inflammatory processes, and behavior mediated by DNA methylation in OCD brain tissues. Notably, the main findings were related to the immune system, reaffirming the current literature findings about its involvement with OCD [104, 105]. It was argued that these changes in DNAm could mediate the action of genetic and environmental factors associated with OCD. Neuroimaging and three of the above-mentioned post-mortem brain studies [77, 91, 97] on OCD patients have consistently shown alterations in the OFC and striatum, *i.e*., brain regions involved in action selection and decision-making, respectively. Another recent study on post-mortem human brain samples was the first to provide evidence of molecular changes in these brain regions, which potentially contribute to such abnormal functioning [106]. This investigation by Piantadosi and his colleagues [106] followed in the wake of the first published post-mortem study on OCD that explored the possible underlying molecular substrates of glutamatergic and GABAergic signalling changes by quantifying gene expression in brain tissue [73]. As reported earlier in this review, decreased PVALB was observed in patients compared to controls, but gene set analyses did not reveal either glutamatergic or GABAergic neurotransmission as a top-altered pathway. The earlier study did not examine the OFC or striatum, which have been more commonly implicated in the OCD disease process [53, 107-111] than the DLPFC [109]. To assess whether glutamatergic and/or GABAergic signalling is altered in these brain regions, the researchers collected grey matter tissue samples from medial OFC (BA 11), lateral OFC (BA 47), head of caudate, and NAC in eight OCD patients and eight matched healthy controls [106]. Quantitative polymerase chain reaction (qPCR) was performed on a panel of transcripts encoding proteins related to excitatory synaptic structure, excitatory synaptic receptors/transporters, and GABA synapses. There were lower levels of multiple glutamatergic transcripts in OCD patients in both the striatum and OFC, with reductions more pronounced in the OFC. In addition to being hampered by a small sample size, another limitation of this study was that it investigated a relatively small subset of transcripts only. Nevertheless, this study provided the first evidence of potential molecular dysfunction in brain regions that have consistently been implicated in neuroimaging studies on OCD. To address the caveats of two of the earlier studies [91, 106], Piantadosi and his colleagues [112] conducted another post-mortem brain study on OCD, using unbiased RNA sequencing to examine the transcriptome of two OFC regions (medial and lateral OFC) and two striatal subregions (CN and NAC) in eight patients with OCD and eight unaffected controls. They focused on two levels of analysis for differentially expressed genes (DEGs), *i.e*., a *global* analysis (from the OCF, CN, and NAC, jointly) and a *regional* analysis (from each brain region considered separately). An investigation of the global differential expression in all regions jointly identified 904 transcripts that were differentially expressed as a function of OCD diagnosis, with most DEGs found in the CN and NAC and not in the OFC. Subsequent gene set enrichment and hierarchical clustering of all differentially expressed transcripts pointed to a hub of pathways that are associated with the glutamatergic synapse and are involved in synaptic neurotransmission. As such, these findings provide additional support for the involvement of striatal and cortical glutamatergic synaptic dysfunction in the pathogenesis of OCD. In terms of quality assessments as per the AXIS tool, the total appraisal score values suggested that the overall quality of the included publications was acceptable and that study designs were appropriate for the aims and objectives set out in their methods sections. In terms of the limitations of the studies included in the review, it may be noted that all were hampered by their sample size. The heterogeneity of OCD cannot be addressed carefully in such small samples. One attempt to overcome this limitation may entail combining diagnostic groups (as was done by Jaffe *et al.* [73]), granting the researchers a much larger sample to interrogate molecular associations. This approach is consistent with the transdiagnostic research framework proposed in the National Institute of Mental Health’s Research Domain Criteria (RDoC), incorporating common symptoms (*e.g*., obsessionality, perfectionism, anxiety, a strong need to control one’s environment and rigidity) that potentially involve shared genetic risk across obsessive syndromes. Another limitation is the relatively narrow selection of brain regions that were investigated. Future work targeting additional brain regions may be required to identify molecular mechanisms involved in OCD. Furthermore, studies investigating regional brain microglial cell and astrocyte activation and protein levels to verify gene expression levels that are associated with the pathophysiology of OCD are lacking. It is unclear whether microscopic alterations in the respective brain regions that were investigated in these cross-sectional studies, represent a primary site or cause of the disorder, are the result of a compensatory mechanism after the disorder developed, or are epiphenomena warranting further study. When conducting OCD post-mortem brain research, maintaining high-quality frozen specimens may be challenging. Important considerations include the rapidity of death (the time between onset of the terminal phase and death) and agonal state, as well as the interval between death and refrigeration of the body (*i.e*., the refrigeration interval) and the time in storage (*i.e*., the period between when autopsy tissue is stored and when it is used). To ensure tissue quality, care should be taken that the interval between death and brain removal prior to freezing or fixation is short, as lengthy post-mortem interval has been shown to affect RNA integrity in microarray experiments, that cerebrospinal fluid (CSF) has acceptable pH (with pH providing a basic estimation concerning agonal state or other variables that can influence tissue quality), and that the RNA integrity number after RNA extraction and preparation is of good quality. Of note also is that in some studies [77], patients were only diagnosed with OCD after death. In terms of best estimated diagnostic procedures, a screening interview by at least one mental health clinician to characterize the OCD symptoms, encompassing psychiatric parameters to the patient during the ante-mortem period, or with a family member or a close caregiver who had regular contact with the deceased, and comprising, for example, the Structured Clinical Interview for DSM [113] and the Yale-Brown Obsessive-compulsive scale (Y-BOCS) (modified to ask the questions about the deceased case), is important [114]. Due to these limitations and challenges associated with post-mortem psychiatry research, such work may be viewed with skepticism. Speculatively, the comparatively low number of publications on post-mortem OCD work (*vs*. schizophrenia, for example) is due to a view of OCD as a less impairing and non-psychotic condition, and therefore less of a research priority. However, the articles included in this systematic review emphasize the potential value of post-mortem brains from well-characterized individuals with OCD for research. Indeed, the development of validated molecular techniques that can be applied to post-mortem tissue has the potential for improved integration of findings from human post-mortem studies with that of animal and cellular models of disease (or of drug effects) [115]. Contributing to data from OCD animal models and from neurocircuitry and neurochemistry studies on humans with OCD which support an association between aberrant activity in CSTC regions and a role for mono-aminergic neurotransmitters as well as the glutamatergic system in OCD, post-mortem studies thus far have provided data on DNA methylation, cellular and molecular alterations, and gene expression profiling in post-mortem tissue of brain areas associated with OCD. Post-mortem investigations, supported by reliable diagnostic procedures and information on comorbid psychiatric and neurological conditions, and on risk factors, such as environmental factors, may provide a strategy to address the caveats in our understanding of the neurobiology of OCD [116-119]. Post-mortem research can potentially contribute to our conceptualisation of psychiatric conditions, by determining, for example, whether shared behaviours (*e.g*., RDoC themes, as noted earlier in the review) across disorders have similar neuropathological underpinnings. Of relevance, although not specifically referring to OCD, is the suggestion that post-mortem research on fresh glial cells (collected soon after brain dissection) can be used to investigate the impact of environmental risk factors *in vitro*, and which may ultimately point to new pathways for interventions [120]. Ultimately post-mortem studies, including single cell genomic research, may facilitate the development and validation of biomarkers in peripheral blood, neuroimaging methods, and discovery of novel targets for therapeutic interventions [120-122]. Finally, any discussion on post-mortem research data would not be complete if the issue of brain donation for neurobiological research was not addressed. The concept of brain donation for scientific research is still largely unknown to the general public. Creating public awareness of 1) the utility and significance of post-mortem material, and 2) the possibility of brain donation for research, is crucial. There have been several initiatives to increase high quality post-mortem psychiatric brains (with comprehensive clinical information) for research purposes, including from OCD cases. These include initiatives of the Netherlands Brain Bank for psychiatry [70, 120] and the Brazilian psychiatric brain bank [123], amongst others, and many of these brain banks operate as members of larger consortia that maintain virtual inventories of their combined holdings. Since the relatively recent initiation of these and other brain banks, the number of registered donors with a psychiatric diagnosis has increased significantly. Interestingly, in a Dutch study that compared recruitment rates across diagnoses (*i.e*., healthy controls, bipolar disorder, major depressive disorder, post-traumatic stress disorder, and OCD), it was found that people with OCD were least likely to register themselves as brain donors. Other findings suggested that face to face recruitment of donors was more successful than postal invitations, and that the likelihood of registering as brain donor significantly increased with age [124]. When initiating a brain bank program in psychiatric patients, consideration of these variables is crucial. Such brain bank resources grant researchers access to brain tissue and clinical information, allowing them to address questions that cannot be answered through other techniques. De Lange [70] has highlighted several questions pertaining to psychiatric disorders in general which have also not yet been addressed by OCD literature, for example, (1) Are the numbers of neuronal or microglial cells altered in specific brain regions? (2) Are the morphology and phenotype of these cells altered in specific brain regions? (3) Is there a change in neuronal function? (4) Are there synaptic abnormalities? (5) Which molecular signaling pathways are affected? In OCD, answers to these questions would be of benefit, contributing knowledge and insight into the underpinnings of this heterogeneous disorder.

## CONCLUSION

In conclusion, this paper systematically reviewed findings from post-mortem brain research in OCD. The very few published post-mortem investigations on OCD were mostly focused on gene expression, providing data on DNA methylation, cellular and molecular alterations, and gene expression profiling in brain areas associated with OCD, thus highlighting the potential value of post-mortem brains from well-characterized individuals with OCD and emphasizing the need for additional work in this area.

## Figures and Tables

**Fig. (1):**
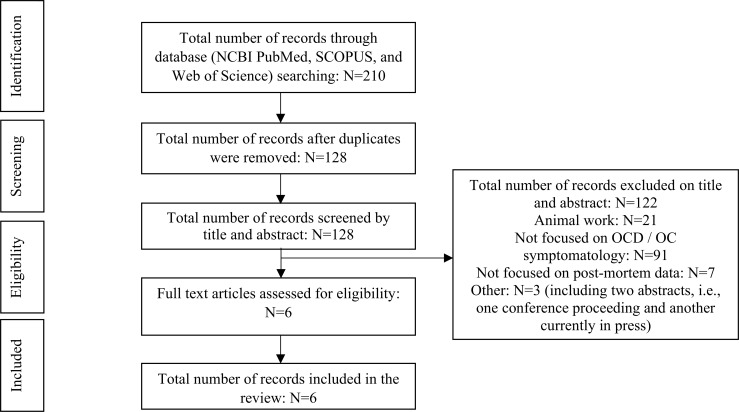
Study selection flow diagram according to the PRISMA statement [71].

**Table 1:** Articles included in the systematic review.

**References**	**Country and Source of Brain Specimens**	**Design**	**Sample Size and Population**	**Diagnostic Method**	**Brain Regions Investigated**	**Main Findings**
Piantadosi *et al.*, 2021 [112]	USA Brain specimens were obtained from the University of Pittsburgh Brain Tissue Donation Program during autopsies conducted by the Allegheny County Medical Examiner’s Office (Pittsburgh, PA).	This study assessed potential transcriptional changes in 3 brain areas (OFC and two striatal regions (caudate nucleus and NAC*) of OCD cases compared to controls. Messenger RNA transcripts from these regions were sequenced.	7 OCD patients, 8 controls	An independent panel of experienced clinicians reviewed findings from structured interviews with relatives, clinical records, toxicology, and neuropathology to make consensus DSM-IV diagnoses. Unaffected controls underwent identical assessments and were determined to be free of any lifetime psychiatric illnesses.	OFC and two striatal regions (caudate nucleus and NAC)	The study data showed that the 904 differentially expressed transcripts showed enrichment for genes involved in synaptic signalling, with the synapse-associated genes displaying lower expression in patients with OCD compared to controls. Cell type fractions of medium spiny neurons were lower, while vascular cells and astrocyte fractions were higher in the brain tissue of OCD patients. It was concluded that the synapse might be considered a “vulnerable molecular compartment” in OCD.
Piantadosi *et al.*, 2021 [106]	USA Same as [112].	This study examined the expression of synaptic genes in post-mortem brain samples of the OFC and striatum from OCD patients and unaffected controls. Quantitative polymerase chain reaction (qPCR) was performed on a panel of transcripts encoding proteins related to excitatory synaptic structure, excitatory synaptic receptors/transporters, and GABA synapses.	8 OCD patients, 8 controls	Same as [112].	Total grey matter tissue samples from medial OFC (BA11), lateral OFC (BA47), head of caudate, and NAC	Patients with OCD had significantly lower levels of several transcripts related to excitatory signaling in cortical and striatal regions compared to controls. The majority of transcripts encoding excitatory synaptic proteins were lower in the OFC but not significantly different in the striatum of OCD patients. These findings were consistent with composite transcript level measures, which revealed that reductions in transcripts encoding excitatory synaptic structure proteins and excitatory synaptic receptors/transporters occurred primarily in the OFC of OCD patients. Transcripts associated with inhibitory synaptic neurotransmission showed minor differences between OCD and control groups.
De Oliveira *et al.*, 2021 [97]	Brazil Brain samples were retrieved from the Sao Paulo Autopsy Service from the University of São Paulo as part of the psychiatric disorders collection of the Biobank for Aging Studies.	In this study, DNA methylation changes and gene expression were evaluated in post-mortem brain tissues of the cortical (anterior cingulate gyrus and OFC) and ventral striatum (NAC, caudate nucleus, and putamen) areas from OCD patients and unaffected controls.	8 OCD patients, 8 controls	Three psychiatrists performed a screening interview encompassing clinical, functional, cognitive, and psychiatric parameters with a family member or close caregiver who had at least weekly contact with the deceased. For individuals with a likely diagnosis of OCD, a second clinical evaluation with the same informant was done for the confirmed diagnosis. The evaluation comprised the Structured Clinical Interview for DSM IV Axis I disorders (SCID) [73], the Yale-Brown Obsessive-compulsive scale (Y-BOCS) [74], and a short version of DY-BOCS [75]. The questionnaires were modified to ask questions about the deceased case. There were thus two assessments for each OCD case.	Cortical (anterior cingulate gyrus and OFC) and ventral striatum (NAC, caudate nucleus, and putamen) areas	Study findings suggested no differentially methylated CpG (cytosine-phosphate-guanine) sites (DMSs) in any brain area of patients with OCD. However, the gene modules generated from CpG sites and protein-protein interaction (PPI) showed enriched gene modules for all brain areas. All brain areas except the NAC presented a predominantly hypomethylation pattern for the differentially methylated regions (DMRs). Although there were common transcriptional factors that targeted these DMRs, their targeted differentially expressed genes differed between the investigated brain areas. The protein-protein interaction network based on methylation and gene expression data reported that all brain areas were enriched for the G-protein signalling pathway, immune response, apoptosis, and synapse biological processes and also presented enrichment of specific signalling pathways. OCD patients and controls did not show significant DNA methylation age differences.
De Oliveira *et al.*, 2019 [77]	Brazil Same as [97].	The researchers applied unbiased stereological tools to estimate post-mortem neuronal density and volume within OFC areas to achieve a clearer understanding of the neurobiological basis of OCD.	7 OCD patients, 7 controls	Same as [97].	The anteromedial (AM), medial orbitofrontal (MO), and anterolateral (AL) areas of the OFC	The study findings suggested statistically significant layer- and region-specific lower neuron densities in the OCD cases that added to a deficit of 25% in the anteromedial and anterolateral and to a deficit of 21% in the orbitofrontal areas of the orbitofrontal cortex OFC), respectively. The volumes of the OFC areas were similar between the OCD and control groups.
Lisboa *et al.*, 2019 [91]	Brazil Same as [97].	The transcriptomes of 3 separate striatal areas (putamen, caudate nucleus, and NAC) were compared between OCD patients and controls.	6 OCD patients, 8 controls	Same as [97].	Three separate striatal areas (putamen, caudate nucleus and NAC)	The study findings suggested that the differentially expressed genes as well as network connectivity deregulation were specific for each of the striatum regions (putamen, caudate nucleus, and NAC) by comparing OCD cases and controls. Some genes that were associated with rare or common variation in published large-scale OCD genomic studies were differentially expressed in specific region comparisons.
Jaffe *et al.*, 2014 [73]	USA Brain specimens were donated through the Offices of the Chief Medical Examiners of the District of Columbia and of the Commonwealth of Virginia, Northern District, to the NIMH Brain Tissue Collection at the NIH in Bethesda, MD.	The researchers measured gene expression in the PFC of cases previously diagnosed with obsessive psychiatric disorders, *i.e*., eating disorders (EDs) and OCD/obsessive-compulsive personality disorder (OCPD) or tics (OCD/OCPD/ Tics), and healthy controls.	15 OCD patients, 15 patients with eating disorders, 16 controls	All cases in the obsessive cohort met DSM-IV criteria for one or more lifetime Axis I diagnoses of an ED (anorexia nervosa, bulimia nervosa, or ED not otherwise specified) and/or OCD, OCPD and/or a tic disorder. Clinical data included family informant interviews with next-of-kin, retrospective psychiatric record reviews, and medical examiner data, including cause/manner of death.	Dorsolateral PFC	The study identified 6 genes that were differentially expressed between ED cases compared with controls. They identified 286 genes that were differentially expressed between OCD cases compared with controls (in both comparisons, the false discovery rate (FDR) was less than 5%). However, none of the clinical risk single nucleotide polymorphisms (SNPs) were among the expression quantitative trait loci (eQTLs), and none were significantly associated with gene expression within the cohort of broadly obsessive psychiatric disorders.

## Data Availability

Not applicable.

## LIST OF ABBREVIATIONS

ACC: Anterior Cingulate Cortex
AL: Anterolateral
AM: Anteromedial
CN: Caudate Nucleus
CSF: Cerebrospinal Fluid
CSTC: Cortico-striato-thalamo-cortical
GBD: Global Burden of Disease
MO: Medial Orbitofrontal
NAC: Nucleus Accumbens
OCD: Obsessive-compulsive Disorder
OFC: Orbitofrontal Cortex
SSRI: Selective Serotonin Reuptake Inhibitor

## STANDARDS OF REPORTING

PRISMA guidelines and methodology were followed.
